# Frequency and Risk Factors for Diuretic Resistance in Patients with Decompensated Heart Failure: A Retrospective Single-Center Study in Western Mexico

**DOI:** 10.3390/medsci14020304

**Published:** 2026-06-11

**Authors:** Leobardo Saúl De la Torre-Cabrales, Sol Ramírez-Ochoa, Gabino Cervantes-Pérez, Berenice Vicente-Hernández, Gabino Cervantes-Guevara, Alejandro Gonzalez-Ojeda, Clotilde Fuentes-Orozco, Francisco Javier Hernandez-Mora, Janet Cristina Vázquez-Beltrán, Mauricio Alfredo Ambriz-Alarcón, Luis Asdruval Zepeda-Gutiérrez, Enrique Cervantes-Perez

**Affiliations:** 1 Departamento de Medicina Interna, Hospital Civil de Guadalajara Fray Antonio Alcalde, Centro Universitario de Ciencias de la Salud, Universidad de Guadalajara, Guadalajara 44280, Mexico; saul_0194@hotmail.com (L.S.D.l.T.-C.); sramirez@hcg.gob.mx (S.R.-O.); gabino-1994@hotmail.com (G.C.-P.); dra.berenicevicente@gmail.com (B.V.-H.); luis-zent@hotmail.com (L.A.Z.-G.); 2 Departamento de Gastroenterología, Hospital Civil de Guadalajara Fray Antonio Alcalde, Guadalajara 44280, Mexico; gabino_guevara@hotmail.com; 3 Departamento de Bienestar y Desarrollo Sustentable, Centro Universitario del Norte, Universidad de Guadalajara, Guadalajara 46200, Mexico; 4 Unidad Biomédica 02, Hospital de Especialidades, Centro Médico Nacional de Occidente, Guadalajara 44350, Mexico; avygail5@gmail.com (A.G.-O.); clotilde.fuentes@gmail.com (C.F.-O.); 5 Departamento de Clínicas de la Reproducción Humana, Crecimiento y Desarrollo Infantil, Centro Universitario de Ciencias de la Salud, Universidad de Guadalajara, Guadalajara 44280, Mexico; frank.gine@gmail.com; 6 Departamento de Obstetricia, Hospital Civil de Guadalajara Fray Antonio Alcalde, Guadalajara 44280, Mexico; 7 Escuela de Medicina, Instituto Politécnico Nacional, Ciudad de Mexico 11340, Mexico; janet.cris.beltran@gmail.com; 8 División de Medicina, Hospital Civil de Guadalajara Fray Antonio Alcalde, Guadalajara 44280, Mexico; mau_ambriz@hotmail.com; 9 Departamento de Disciplinas Filosóficas, Metodológicas e Instrumentales, Centro Universitario de Ciencias de la Salud, Universidad de Guadalajara, Guadalajara 44340, Mexico; 10 Departamento de Clínicas, Centro Universitario de Tlajomulco, Universidad de Guadalajara, Tlajomulco de Zuñiga 45641, Mexico

**Keywords:** heart failure, diuretic resistance, chronic kidney disease

## Abstract

Background/Objectives: Diuretic resistance is a recognized complication in patients with heart failure (HF) and is associated with worse clinical outcomes; however, information regarding its frequency and associated factors in hospitalized patients in Mexico is limited. This study aimed to describe the frequency of diuretic resistance in patients hospitalized with HF in a hospital unit in western Mexico and to identify factors associated with diuretic resistance. Methods: This retrospective study used data obtained from clinical records. Patients older than 18 years with decompensated HF whose complete clinical records included the variables of interest were included. Patients were classified according to the presence or absence of diuretic resistance. Bivariate and multivariate analyses were performed to evaluate factors associated with diuretic resistance. Results: A total of 76 patients were analyzed, and the frequency of diuretic resistance was 35.5% (*n* = 27). In bivariate analysis, type 2 diabetes mellitus, chronic kidney disease, elevated creatinine, urea, blood urea nitrogen (BUN), and urine protein levels, decreased glomerular filtration rate (GFR) and serum albumin, and prior treatment with nonsteroidal anti-inflammatory drugs (NSAIDs) and angiotensin-converting enzyme inhibitors/angiotensin II receptor antagonists (ACEI/AARII) were significantly associated with diuretic resistance. In the multivariate logistic regression model, prior ACEI/AARII treatment, history of type 2 diabetes mellitus, BUN levels, and serum albumin levels remained independently associated with diuretic resistance classification. Conclusions: Diuretic resistance was frequent in this cohort of patients hospitalized with decompensated heart failure, and several clinical and biochemical factors were independently associated with its occurrence. These findings may help identify patients at higher risk of diuretic resistance, although they should be confirmed in future prospective studies.

## 1. Introduction

Heart failure (HF) is a clinical syndrome primarily defined by signs and symptoms resulting from a cardiac abnormality, whether structural or functional, such as elevated natriuretic peptide levels and/or objective evidence of pulmonary or systemic congestion [1].

Globally, HF is considered a pandemic, with data from 2017 indicating that approximately 64.3 million patients are affected worldwide [1]. In 2019, based on data obtained from the ATLAS project, the prevalence was estimated to be 17 per 1000 inhabitants across approximately 13 European countries [2]. In 2012, the American Heart Society, based on data collected between 2015 and 2018, estimated that more than 5.7 million Americans over the age of 20 had HF, with a prevalence of 2.4%, and projected that it would increase to as high as 3% by 2030 [3,4].

The prevalence of HF ranges from 1–3% in adult patients in developed countries and increases to more than 10% and 30% in individuals over 70 and 85 years of age, respectively [5]. Some studies report an incidence of HF of 1–4 per 1000 people/year [6,7]. The Rotterdam study estimated that the lifetime risk of heart failure for men and women is 33% and 28%, respectively [8]. In general, a higher incidence has been reported in men than in women (15 and 12 per 1000 people/year, respectively) [9]. Over the past 20 years, survival in patients with HF has improved due to the development of pharmacological and nonpharmacological therapies. After the diagnosis of HF, the estimated survival rate is 72–75% at 1 year and 35–52% at 5 years [6]. The in-hospital mortality rate is 4–10% [10]. Patients newly diagnosed with acute decompensation have higher in-hospital mortality rates than those with previously known chronic heart failure; however, they have lower post-discharge mortality and rehospitalization rates [11,12]. Patients hospitalized for HF have the highest 30-day rehospitalization rates [13]. Moreover, nearly half of these patients are re-hospitalized within 1 year, with most of these hospitalizations being due to non-cardiovascular causes [14].

Decompensated HF is the most common cause of acute heart failure, accounting for 50–70% of patients presenting to emergency departments. The main underlying mechanism is left ventricular dysfunction with renal retention of sodium and water; thus, fluid accumulation increases intraventricular pressure and results in a gradual onset of symptoms, while cardiac output may be normal or decreased [15,16].

The biochemical parameters used to confirm the presence of decompensated heart failure include B-type natriuretic peptide (BNP), N-terminal pro B-type natriuretic peptide (NT-proBNP), and mid-regional pro atrial natriuretic peptide (MR-proANP) [17,18].

BNP and NT-proBNP have high sensitivity for ruling out acute HF [19]. The BNP cutoff values for the diagnosis of decompensated HF are 100 pg/mL (sensitivity 95%, specificity 63%, positive predictive value 67%, negative predictive value 94%) and 400 pg/mL to improve specificity and positive predictive value (sensitivity 85%, specificity 86%, positive predictive value 85%, and negative predictive value 86%); the cutoff value for NT-proBNP is 300 pg/mL (sensitivity 99%, specificity 43%, positive predictive value 64%, and negative predictive value 98%) [20]. There are age-specific cutoff values for NT-proBNP, with values of 450, 900, and 1800 pg/mL for individuals under 50 years, 50–75 years, and over 75 years, respectively [17]. Several factors can influence natriuretic peptide levels, including age, sex [21], body mass index, kidney function, and medication use [22].

Diuretic resistance is an important challenge in the management of patients with acute heart failure, occurring in approximately one in three patients with HF [23]. Several causes have been described, including inadequate diuretic dosing, intestinal edema, chronic kidney disease, hypotension at presentation, renal hypoperfusion, concomitant use of NSAIDs, hyponatremia, hypochloremia, acidic urinary pH, hypoalbuminemia, metabolic alkalosis, and inadequate blockade of the renin–angiotensin–aldosterone system, as well as other unidentified factors [23]. There is no single definition to describe this condition; however, it is generally characterized as the inability to achieve adequate decongestion despite appropriate and escalating doses of diuretics [24].

Various therapeutic strategies exist for patients with diuretic resistance; however, there is no clear consensus regarding optimal management. Most experts recommend delaying combination therapy until loop diuretics have been optimized, since combined use, despite its physiological rationale, increases the risk of severe electrolyte disturbances [23,24,25]. Diuretic resistance has also been associated with longer hospital stays, prolonged symptom duration, and increased healthcare costs per hospitalization and per day of treatment [23]. In this exploratory study, we aimed to describe the frequency of diuretic resistance in patients hospitalized with HF in a hospital unit in western Mexico and to identify factors associated with diuretic resistance in this patient population.

## 2. Materials and Methods

For this retrospective study, the records of patients hospitalized in the Internal Medicine Department of the Hospital Civil de Guadalajara “Fray Antonio Alcalde” were reviewed. The following inclusion criteria were considered: patients of both sexes older than 18 years with a diagnosis of decompensated heart failure from January 2020 to October 2022. The diagnosis had to meet the Framingham diagnostic criteria [9], as well as BNP criteria within the cutoff values for HF, defined as follows: hospitalized patients with BNP > 400 pg/mL or NT-proBNP > 450 pg/mL if <50 years, >900 pg/mL if 50–75 years, or >1800 pg/mL if >75 years. In addition, patients could not meet any of the following exclusion criteria: hospital records lacking variables required to determine response to diuretics, incomplete clinical studies with respect to the variables of interest, or death within the first 72 h after admission. To identify factors associated with diuretic resistance, clinical and biochemical variables at admission were collected.

The diuretic dose administered during the first 72 h was also recorded, as was the amount of diuresis obtained according to the administered diuretic dose, to classify whether the patient met the definition of diuretic resistance. To establish whether the patient presented diuretic resistance, the definition proposed by Lu et al. [26] was used. Given the lack of a universally accepted operational definition of diuretic resistance, this definition was selected because it provides a reproducible criterion based on both diuretic dose and urinary output, is supported by previous reviews on the pathophysiology and clinical evaluation of diuretic response [24,27], and has been previously applied in a predictive model of diuretic resistance. Diuretic resistance was defined as insufficient decongestion despite oral or intravenous (IV) furosemide >160 mg or >80 mg in one day and/or IV torsemide >80 mg or >40 mg in one day and/or IV bumetanide >4 mg in one day, with urinary output <750 mL/24 h.

Once diuretic resistance was established, the sample was divided into two groups (patients with HF and diuretic resistance and patients with HF without diuretic resistance) for comparison. To store information, a database was created in Microsoft Excel. GraphPad Prism (version 9.3.1.471, GraphPad by Dotmatics, Boston, MA, USA) was used for statistical analysis.

For descriptive statistics of the qualitative variables, both absolute and relative frequencies were calculated, and the data are presented as percentages and proportions; likewise, the proportions of dichotomous categorical variables were arranged in a 2 × 2 contingency table and compared using the chi-square test. For quantitative variables, measures of central tendency and dispersion (mean, median, and standard deviation) were used. Regarding inferential statistics for quantitative variables, normality was first assessed using the D’Agostino–Pearson and Kolmogorov–Smirnov tests. Once the results were obtained, comparisons between unpaired groups were performed using Student’s t test for parametric data and the Mann–Whitney U test for nonparametric data.

Multivariate analysis was performed using binary logistic regression with a backward stepwise entry model, likelihood ratio testing, and the Hosmer-Lemeshow goodness-of-fit test, using all statistical and clinical variables that were significant in bivariate analysis. In the logistic regression model, the absence of diuretic resistance was used as the modeled outcome. Therefore, the reported odds ratios (ORs) represent the odds of belonging to the non-resistant group. For categorical variables, ORs were interpreted according to the presence versus absence of the evaluated factor, whereas for continuous variables, ORs represent the change in odds associated with each one-unit increase in the variable. ORs and their corresponding 95% confidence intervals (CIs) were calculated. In all analyses, *p* values < 0.05 were considered statistically significant. The study was conducted in accordance with the Declaration of Helsinki. The Clinical Research and Bioethics Committee of the Hospital Civil de Guadalajara “Fray Antonio Alcalde” approved this study (R-98/23), and the requirement for informed consent for this retrospective analysis was waived.

## 3. Results

From the review of hospital records, 220 patient records were identified, of which 141 were excluded because they were incomplete, while 3 patient records were excluded because the patients died within 72 h after admission. Thus, 76 patients met the inclusion criteria, did not meet any exclusion criteria, and were included in the study. Among these 76 patients, 35.5% (*n* = 27) were resistant to diuretics. The baseline demographic characteristics of the patients are presented in Table 1.

To identify factors associated with diuretic resistance in the included patients, an analysis of the collected clinical and laboratory characteristics was performed, and the results are shown in Table 2.

Multivariate analysis was performed to determine the factors independently associated with diuretic resistance classification, using absence of diuretic resistance as the modeled outcome. Variables that were significant in the bivariate analysis were included in the multivariate model, including history of diabetes mellitus, chronic kidney disease (CKD), creatinine, urea, blood urea nitrogen (BUN), albumin, glomerular filtration rate (GFR), and prior treatment with nonsteroidal anti-inflammatory drugs (NSAIDs) and ACEI/AARII.

Four variables were retained in the final multivariate logistic regression model: history of type 2 diabetes mellitus, BUN levels, serum albumin levels, and prior ACEI/AARII treatment (Table 3). Because the absence of diuretic resistance was used as the modeled outcome, the direction of the odds ratios should be interpreted as the odds of belonging to the non-resistant group.

In the multivariate logistic regression model, the absence of diuretic resistance was used as the modeled outcome. Therefore, ORs below 1 indicate lower odds of belonging to the non-resistant group, whereas ORs above 1 indicate higher odds of belonging to the non-resistant group. For continuous variables, ORs represent the change in odds associated with each one-unit increase in the variable.

## 4. Discussion

In our study, the frequency of diuretic resistance in patients with HF was 35.5%, which is consistent with data reported in the literature, where diuretic resistance occurs in approximately one in three patients with decompensated heart failure [23]. Notably, the results from the REDIHF registry reported a prevalence of 21.2% [28]. The absence of a universally accepted definition of diuretic resistance should be considered when comparing our findings with those of previous studies. Different operational criteria based on diuretic dose, urinary output, natriuretic response, weight loss, or clinical decongestion may contribute to variability in the reported frequency of diuretic resistance. Therefore, although our findings are consistent with previous reports suggesting that diuretic resistance is common in patients with decompensated HF, direct comparisons across studies should be made with caution. In addition, owing to the exploratory nature of our study and limitations in the electronic clinical records, the prevalence of diuretic resistance could not be determined, and the measure reported in this study corresponds to frequency.

Regarding the patients’ clinical history, a statistically significant association was observed between chronic kidney disease and diuretic resistance in the bivariate analysis (OR = 9; 95% CI: 2.5–27.5), as well as between a history of type 2 diabetes mellitus and diuretic resistance (OR = 23; 95% CI: 3.3–246). In the multivariate model, history of type 2 diabetes mellitus remained independently associated with diuretic resistance classification. Lu et al. [26] conducted a single-center, retrospective, observational study to evaluate factors associated with the development of diuretic resistance during hospitalization. They included 15,444 patients without diuretic resistance and 3383 patients who developed diuretic resistance, reporting a frequency of 17.97%. They observed a higher frequency of diuretic resistance in women, as well as a higher prevalence of pneumonia, type 2 diabetes mellitus, and kidney disease among patients with diuretic resistance, with all factors showing statistically significant differences. In our study, significant differences in clinical history were observed only for type 2 diabetes mellitus (in both bivariate and multivariate analyses) and chronic kidney disease (only in the bivariate analysis) in relation to diuretic resistance. However, the sample size was limited, and these findings should be interpreted with caution. Although these results suggest that certain clinical conditions may be associated with diuretic resistance, further studies are required to confirm these observations.

Patients with diuretic resistance had significantly higher levels of creatinine, urea, and BUN (Table 2). In addition, these patients had a lower glomerular filtration rate (37 vs. 69 mL/min/1.73 m^2^, *p* = 0.0001). In the multivariate model, BUN remained independently associated with diuretic resistance classification. These results are consistent with findings reported in the literature [26,28,29], which suggest that impaired renal function may be associated with diuretic resistance.

Patients with diuretic resistance had lower mean serum albumin levels than those without diuretic resistance (2.59 vs. 3.01 g/dL, *p* = 0.0069). In the multivariate model, serum albumin remained independently associated with diuretic resistance classification. These findings are consistent with those reported in the literature [26,28,29]. A possible explanation is that furosemide binds primarily to albumin until it reaches its site of action; therefore, decreased albumin levels may limit its availability [24]. Patients with diuretic resistance also had higher levels of proteinuria (72.5 vs. 38.78 mg/dL), a variable that has not been widely described as associated with diuretic resistance. Although this finding may be related to the degree of renal dysfunction observed in these patients, it should be interpreted with caution and confirmed in future studies with larger sample sizes.

In our study, no significant differences were observed in serum sodium and chloride levels between groups, which contrasts with some reports in the literature [26,30,31]; however, this discrepancy may be explained by the limited sample size of our study.

Previous use of furosemide was also analyzed as a factor associated with diuretic resistance; however, no statistically significant differences were observed. In contrast, prior use of nonsteroidal anti-inflammatory drugs (NSAIDs) was significantly more frequent in the group with diuretic resistance. This finding may be related to the greater degree of renal dysfunction observed in these patients and is consistent with previous reports [28,29].

Another variable analyzed was prior treatment with angiotensin-converting enzyme inhibitors/angiotensin II receptor antagonists (ACEI/AARII). In the group with diuretic resistance, 92.5% of patients reported prior use, compared with 60% in the group without diuretic resistance (OR = 8.1; 95% CI: 1.7–37.7). In the multivariate model, prior ACEI/AARII treatment was associated with lower odds of belonging to the non-resistant group, consistent with its higher frequency among patients with diuretic resistance in the bivariate analysis. Although RAAS blockade is generally expected to improve neurohormonal modulation in HF, the relationship between RAAS activity, RAAS-modifying therapy, renal hemodynamics, and diuretic response is complex [25,28,29,32,33,34]. In susceptible patients with decompensated HF and renal hypoperfusion, changes in efferent arteriolar tone and intraglomerular pressure may influence renal function and diuretic responsiveness [32,33,34]. Alternatively, prior ACEI/AARII use may have been a marker of more advanced cardiovascular or renal disease rather than a direct cause of diuretic resistance. Because of the retrospective design and limited sample size, this association should be interpreted cautiously and considered hypothesis-generating. No significant differences in mortality were observed between groups. Although previous studies have reported an association between diuretic resistance and increased mortality [29], the retrospective design of our study and the lack of post-discharge follow-up limit the interpretation of this finding.

The multivariate logistic regression model should be interpreted according to the coding of the dependent variable. Because the absence of diuretic resistance was used as the modeled outcome, the reported ORs represent the odds of belonging to the non-resistant group. Thus, the ORs below 1 observed for T2DM, BUN, and prior ACEI/AARII treatment indicate lower odds of the non-resistant outcome, whereas the OR above 1 observed for serum albumin indicates higher odds of the non-resistant outcome with increasing albumin levels. These findings are directionally consistent with the bivariate results, in which patients with diuretic resistance had a higher frequency of T2DM and prior ACEI/AARII use, higher BUN levels, and lower serum albumin levels. Given the retrospective design and limited sample size, these associations should be interpreted as exploratory and should not be considered evidence of causality.

Our study has several limitations. First, the sample size was relatively small, and all patients were from a single tertiary referral hospital, which may limit the generalizability of the findings. Second, because this was a retrospective study based on clinical records, a substantial number of initially identified records had to be excluded due to incomplete information. This may have introduced selection bias and may limit the representativeness of the analyzed cohort. Third, the retrospective design precludes establishing causal relationships; therefore, the associations observed in this study should be interpreted with caution and confirmed in prospective studies. Finally, the available medical records did not allow long-term follow-up after hospital discharge. Nevertheless, information on diuretic resistance in Mexican populations remains limited. Therefore, this study provides preliminary data that may support future larger prospective studies designed to confirm these findings.

## 5. Conclusions

The frequency of diuretic resistance in patients with HF in our study was 35.5%. Several clinical and biochemical variables were associated with diuretic resistance in bivariate analysis, including a history of type 2 diabetes mellitus and chronic kidney disease; increased creatinine, urea, BUN, and urinary protein levels; decreased glomerular filtration rate (GFR) and serum albumin; and prior treatment with NSAIDs and ACEI/AARII. These findings should be interpreted with caution and confirmed in future prospective studies.

## Figures and Tables

**Table 1:** Sociodemographic characteristics of the study population.

		Resistance to Diuretics (*n* = 27)		No Resistance to Diuretics (*n* = 49)		Odds Ratio (CI 95%)	*p*
		Mean	SD	Mean	SD		
**Age**		55.48	9.41	52.88	9.40	-	0.1963
		No.	%	No.	%		
**Gender**	Male	13	48.15	29	59.18	0.6404 (0.2454–1.6270)	0.3544
	Female	14	51.85	20	40.82		
**T2DM**	Yes	26	96.30	26	53.06	23 (3.348–246.2)	0.0001
	No	1	3.70	23	46.94		
**Arterial Hypertension**	Yes	22	81.48	31	63.27	2.5550 (0.8052–6.961)	0.0980
	No	5	18.52	18	36.73		
**Smoking**	Yes	10	37.04	25	51.02	0.5647 (0.2315–1.430)	0.2418
	No	17	62.96	24	48.98		
**Chronic Kidney Disease**	Yes	12	44.44	4	8.16	9 (2.516–27.59)	0.0002
	No	15	55.56	45	91.84		
**Thyroid disease**	Yes	4	14.81	3	6.12	2.6670 (0.6609–11.13)	0.2098
	No	23	85.19	46	93.88		

SD = standard deviation, CI = confidence interval, T2DM = type 2 Diabetes Mellitus.

**Table 2:** Clinical and biochemical characteristics.

		Resistant to Diuretics (*n* = 27)		No Resistance to Diuretics (*n* = 49)		Odds Ratio (CI 95%)	*p*
		Mean	SD	Mean	SD		
**Hemoglobin (g/dL)**		11.65	1.97	12.92	3.20	-	0.0660
**Creatinine (mg/dL)**		2.37	1.16	1.52	1.12	-	0.0002
**Urea (mg/dL)**		92.70	41.75	71.69	41.65	-	0.0171
**BUN (mg/dL)**		43.27	19.49	33.46	19.43	-	0.0171
**Sodium (mEq/L)**		134.60	6.93	135	5.91	-	0.3999
**Chlorine (mEq/L)**		103	8.23	99.53	7.41	-	0.0618
**Albumin (g/dL)**		2.59	0.68	3.01	0.60	-	0.0069
**Proteins in urine (mg/dL)**		72.59	75.58	38.78	52.90	-	0.0084
**Urinary pH**		5.74	0.68	5.81	0.84	-	0.8891
**GFR (mL/min/1.73 m^2^)**		37.26	23.48	69.92	34.62	-	0.0001
**Systolic arterial Pressure (mmHg)**		143.5	36.04	132.4	33.53	-	0.1838
**Previous doses of furosemide (mg)**		20.74	33.96	18.78	36.61	-	0.6911
		No.	%	No.	%		
**Previous treatment with furosemide**	Yes	8	29.63	12	24.49	1.298 (0.4245–3.480)	0.6263
	No	19	70.37	37	75.51		
**Previous treatment with NSAID**	Yes	27	100	1	2.04	0 (118.2–0)	<0.0001
	No	0	0	48	97.96		
**Prior treatment with ACEI/AARII**	Yes	25	92.59	26	60.47	8.173 (1.798–37.73)	0.0033
	No	2	7.41	17	39.53		
**Death**	Yes	3	11.11	6	12.24	0.8958 (0.2295–3.350)	0.8836
	No	24	88.89	43	87.76		

NSAID = nonsteroidal anti-inflammatory drugs, ACEI/AARII = angiotensin-converting enzyme inhibitor/angiotensin II receptor antagonist, SD = standard deviation, CI = confidence interval, BUN = blood urea nitrogen.

**Table 3:** Multivariate logistic regression model with absence of diuretic resistance as the modeled outcome.

Variables	Odds Ratio for Non-Resistance (CI 95%)	*p*
T2DM	0.024 (0.002–0.364)	0.007
BUN	0.969 (0.938–1)	0.050
Albumin	4.875 (1.591–14.933)	0.006
Prior treatment with ACEI/AARII	0.060 (0.008–0.454)	0.006

CI = Confidence Interval, ACEI/AARII = angiotensin-converting enzyme inhibitor/angiotensin II receptor antagonist, BUN = Blood Urea Nitrogen, T2DM = type 2 Diabetes Mellitus.

## Data Availability

The data presented in this study are available on request from the corresponding author due to privacy reasons.

## Abbreviations

The following abbreviations are used in this manuscript:

HF	Heart failure
GFR	Glomerular filtration rate
NSAID	Nonsteroidal anti-inflammatory drug
ACEI/AARII	Angiotensin-converting enzyme inhibitor/angiotensin II receptor antagonist
BNP	B-type natriuretic peptide
MR-proANP	Mid-regional pro atrial natriuretic peptide
IV	Intravenous
OR	Odds ratio
CI	Confidence interval
SD	Standard deviation
T2DM	Type 2 diabetes mellitus
BUN	Blood urea nitrogen
CKD	Chronic kidney disease
